# The association of patient and trauma characteristics with the health-related quality of life in a Dutch trauma population

**DOI:** 10.1186/s13049-017-0375-z

**Published:** 2017-04-14

**Authors:** Amy Gunning, Mark van Heijl, Karlijn van Wessem, Luke Leenen

**Affiliations:** Department of Trauma Surgery, University Medical Center Utrecht, Suite: G04.228, Heidelberglaan 100, 3584 CX Utrecht, The Netherlands

**Keywords:** Trauma patients, Quality of life, Level I trauma population, Trauma patient outcome, Factors associated with functional outcome, Nonfatal outcome, Trauma centre performances, Benchmarking trauma systems

## Abstract

**Background:**

It is suggested in literature to use the Health Related Quality of Life (HRQoL) as an outcome indicator for evaluating trauma centre performances. In order to predict HRQoL, characteristics that could be of influence on a predictive model should be identified. This study identifies patient and injury characteristics associated with the HRQoL in a general trauma population.

**Methods:**

Retrospective study of trauma patients admitted from 1st January 2007 through 31th December 2012. Patients were aged ≥18 years and discharged alive from the level I trauma centre. A combined health survey (SF-36 and EQ-5D) was sent to all traceable patients. The subdomain outcomes and EQ-5D index value (EQ-5Di) were compared with the reference population. A linear regression analysis was performed to identify parameters associated parameters with the HRQoL outcome.

**Results:**

A total of 1870 patients were included for analyses. Compared to the eligible population, included patients were significantly older, more severely injured, more often admitted in the ICU and had a longer admission duration.

The SF-36 and EQ-5Di were significantly lower compared to the Dutch reference population.

The variables age, Injury Severity Score, hospital length of stay, ICU length of stay, Revised Trauma Score, probability of survival, and severe injury to the head and extremities were associated with the HRQoL in the majority of the subdomains.

**Discussion:**

In order to use HRQoL as an indicator for trauma centre performances, there should be a consensus of the ideal timing for the measurement of HRQoL post-injury and the appropriate HRQoL instrument. Furthermore, standardised HRQoL outcomes must be developed.

**Conclusion:**

This study revealed eight factors (described above) which could be used to predict the HRQoL in trauma patients.

## Background

Mortality rates in trauma patients significantly decreased worldwide after trauma system implementation [1–4]. More severely injured patients survive and because of this nonfatal outcomes become more in demand [5]. That is why outcomes should not only be measured in terms of mortality, but also in morbidity [6]. Assessment of health related quality of life (HRQoL) is important in order to evaluate the success of treatment and to identify areas in which improvement is required [7]. Several studies have quantified the impact of the injuries by measuring HRQoL in these patients [8–11]. It has previously been suggested that more research is necessary for these outcomes to be used for evaluating and benchmarking trauma centre performances [5]. The HRQoL should be measured a significant time after the injury for a valid healing time frame.

Trauma centre performance is currently measured with the Trauma Injury Severity Score method and compared to an international standard [12]. In order to compare the quality of life between trauma patients HRQoL should be standardised and predicted HRQoL compared with the observed HRQoL. The first step in order to calculate these HRQoL outcome predictions is to identify patient and injury characteristics significantly associated with HRQoL. Subsequently standardised HRQoL outcomes must be developed in order to compare observed outcomes with predicted outcomes.

The objective of this study was to identify patient and injury characteristics associated with the HRQoL in a general trauma population.

## Methods

### Study design

This study was performed using the institutional trauma registry of the University Medical Center Utrecht (UMCU). UMCU is a level I trauma centre in the centre of the Netherlands. The UMCU officially became a level I trauma centre in 2000. Annually, 35,000 patients are admitted at the UMCU, of which 1300 trauma patients and 375 severely injured patients (Injury Severity Score [ISS] >15). The trauma centre covers the central region of the Netherlands with a service area of 1,500 km^2^ and approximately 1.3 million residents. Four Level II and III trauma centres are connected to this network. The longest distance between the centres is approximately 50 km.

All trauma patients directly admitted from the Emergency Department are registered in the institutional trauma registry. All patient characteristics are prospectively registered in this registry.

Ethics approval was given by the Medical Ethics Committee of the UMCU (Reference number 12–365).

### Patients

All trauma patients admitted between January 1, 2007 and December 31, 2012 were retrospectively selected from the institutional trauma registry. Patients aged 18 years and older at time of admission and alive at time of discharge were included in the study. All patients invited to participate in the study had a follow up period of at least one year.

The data from patients transferred from a hospital abroad, discharged to another hospital and patients living abroad were excluded.

### Data

Data of baseline characteristics were collected from the regional trauma registry and electronic medical records. The collected data were age, gender, type of injury, Glasgow Coma Scale (GCS), systolic blood pressure (SBP), respiratory rate (RR), Abbreviated Injury Scale (AIS) score (version 2005), ISS, hospital length of stay (H-LOS) Intensive Care Unit length of stay (ICU-LOS), and ICU admission.

The Revised Trauma Score (RTS) was calculated from the GSC, SBP, and RR, according to the formula by Champion et al. [13]. The ISS is calculated from the AIS and represents the severity of all injuries. Multitrauma patients were considered severely injured patients with an ISS ≥16. A severe injury was considered an injury with an AIS ≥3.

The probability of survival for each individual subject was calculated, as previously described [14].

### Follow-up

The basic municipal registry and the electronic medical records were consulted to see whether patients were alive. The addresses of all included patients were retrieved from the electronic patient data management system. All patients received a health survey by mail. If patients consented to participate, they filled out the survey online or returned the form by mail. The surveys were sent from August 2013 until December 2013, to ensure at least 1 year of follow up in all patients. Non-responders were contacted by mail and telephone after 1 month.

### Health related quality of life

Numerous Health Related Quality of Life (HRQoL) instruments have been used in trauma patients. For the purpose of this study two generic health instruments were selected which are currently widely used in trauma patients. The Short Form (36) Health Survey (SF-36) version 2 [15, 16] and the EuroQol 5 Dimensions (EQ-5D) [17]. The SF-36 yields eight multi-item scales that assess different subdomains of functional and emotional health and well-being. The score ranges from 0 to 100 per subdomain; the higher the score the better the outcome. The subdomain ‘physical functioning’ scores the performance of physical activities. ‘Social functioning’ is the subdomain score for interference due to emotional and physical problems with normal social activities. ‘Role-physical’ grades the limitations in daily activities as a result of physical health, and ‘role-emotional’ measures the problems of daily activities as a result of emotional problems. The subdomain ‘mental health’ determines the psychological distress and well-being and ‘vitality’ measures energy and fatigue. The restrictions due to pain were assessed in the ‘bodily pain’ subdomain and the ‘general health’ evaluates the personal health. These subdomains can be reduced to a norm-based summary score, the ‘physical component summary’ (PCS) and a ‘mental component summary’ (MCS). This score has a mean of 50 and a standard deviation of ten.

The index score for EQ-5D (EQ-5Di) ranges from −0.33 to 1.00. One indicates a patient with the best health status, zero indicates a HRQoL comparable to death, and a negative score represents a health status worse than death [17]. The latter health status means a condition in which the patient is completely dependent on others. The patient is unable to walk, to dress, to feed themselves or participate in any activity, and sustain extreme pain or discomfort and depression or anxiousness. The EQ-5D consists of six dimensions: ‘mobility’, ‘self-care’, ‘usual activities’, ‘pain/discomfort’, and ‘depression/anxiety’. All the questions have five response options: no problems, slight problems, moderate problems, severe problems, and extreme problems or unable to. Moreover, the EQ-5D can be dichotomised into ‘problems’ versus ‘no problems’. Problems refer to the four response options: slight, moderate, severe and extreme problems [18].

### Statistical analysis

In the dataset GCS, SBP, and RR were missing in respectively 32%, 29%, and 62% of the cases. In order to calculate survival probabilities for each individual patient, the missing data of the GCS, SBP, and RR were imputed with multiple imputation methods (five datasets). A previous study [19], using the same patient cohort as used in this study, demonstrated that missing data in this population was predominantly found in the less severe injured patients. Moreover, this study showed that multiple imputation did not alter the means of the parameters. The variables in the imputation regression model were the eye, motor, and verbal component of the GCS, SBP, RR, ISS, gender, age, and severity of head injury [20, 21].

The mean scores were calculated with standard deviations for all eight SF-36 subdomains. The mean for each subdomain was compared with the mean score in the Dutch reference population [22]. The Dutch reference population consisted of a sample of Dutch households drawn at random from the national telephone registry. The sample used was limited to individuals 16 years of age and older [22]. To calculate the EQ-5Di, a culture dependent tariff, a country specific conversion formula, is used. The Dutch tariff was used for the calculation of the EQ-5Di in this study. In addition, the EQ-5Di scores were compared with the Dutch reference population. Though this was performed with the EQ-5Di scores, calculated with the United Kingdom (UK) tariff, because there was no Dutch tariff available when the reference scores were established [12, 18]. The outcome of the EQ-5D was dichotomised into ‘problems’ versus ‘no problems’ [18].

Baseline characteristics of the eligible population were compared with the participating population (Table 1). Continuous variables were compared with the Independent-Samples *T* test or Mann-Whitney *U* test, and categorical variables were compared with the Chi-square test. Means are presented with standard deviations and medians with interquartile ranges.

**Table 1:** Patient characteristics eligible versus included population

	Eligible population	Included population
Number of patients	4373	1870
Age	51 (20.1)	54 (18.4) ‡
Gender		
Male	2878 (66)	1178 (63) ‡
Female	1495 (34)	692 (37) ‡
Injury type		
Blunt	4104 (94)	1777 (95)
Penetrating	269 (6)	93 (5)
ISS		
Mean	12.0 (9.5)	13.1 (9.9) ‡
Median	9 (5–17)	10 (5–18) ‡
Multitrauma ISS ≥ 16	1327 (30)	656 (35) ‡
AIS head ≥3	1153 (26)	568 (30) ‡
AIS face ≥3	118 (3)	58 (3)
AIS thorax ≥3	791 (18)	346 (19)
AIS abdomen ≥3	225 (5)	98 (5.2)
AIS extremities ≥3	1129 (26)	518 (28)
H-LOS		
Mean	12 (17.0)	13 (17.7) ‡
Median	6 (3–13)	7 (3–15) ‡
ICU-LOS		
Mean	1 (7.7)	2 (5.3) ‡
Median	NA	NA
ICU admission	689 (16)	336 (18) ‡
Probability of survial^a^		0.98 (0.94–0.99)
Probability of survival <0.50		58 (3)

‡ *p* < 0.05

^a^Mean is presented with standard deviation; median is presented with interquartile range

The patient and trauma characteristics included to evaluate associations with the HRQoL were: age, gender, RTS, ISS, type of injury, multitrauma, severe injury (AIS ≥ 3) for all AIS regions except external, probability of survival, ICU admission, ICU-LOS, and H-LOS. Age was used as a continuous variable (age) and as a categorical variable (age categories). The ‘age categories’ were derived according to the age categories used in the Dutch reference population study [22].

A linear regression analysis was performed to demonstrate the association between the patient characteristics and the subdomain outcomes of the SF-36 and the EQ-5Di value. The continuous scales of the SF-36 and EQ-5Di were used in order to differentiate between the severity of the impact on the HRQoL. The associations were reported with unstandardised coefficients and their level of significance.

A *p*-value lower than 0.05 was considered statistically significant. Due to multiple hypotheses, *p*-values between 0.01 and 0.05 should be interpreted with caution All statistical analyses of the data were performed with SPSS Statistics Version 22.0 (IBM Corp., Armonk, NY) for Windows.

## Results

### Patients

**Fig. 1:**
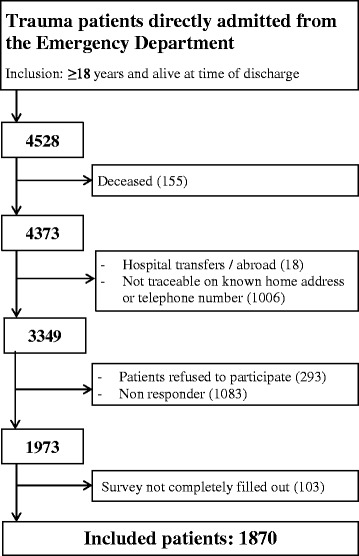
Flowchart

A total of 4528 patients, aged 18 years or older were discharged alive from the trauma hospital of the UMCU through the years 2007 to 2012. After verifying the vital status of the patients, sending the survey by surface mail, and contacting the patient by telephone, 1973 patients (59% of the traceable patients) returned the health survey. Patients who did not completely filled out health surveys were excluded from analysis. A total of 1870 patients were included for the analysis. An overview of the flow of the included number of analysed patients is presented in Fig. 1.

Compared to the eligible population, the included patients were significantly older, were longer admitted in the hospital and more often admitted in ICU. They also were more severely injured and had in particular more severe head injuries. The characteristics of eligible and included population were outlined in Table 1.

### HRQoL outcomes

All mean scores of the eight subdomains of the SF-36 were significantly lower compared to the Dutch reference population [22]. The largest difference between the study and reference population was in the ‘role physical’ domain, followed by the domains ‘general health’ and ‘social function’. The least difference was within the domain ‘bodily pain’ and ‘mental health’. The mean PCS and MCS score were 45.6 and 47.2 respectively (Table 2).

**Table 2:** Mean (SD) SF-36 and EQ-5D outcomes in comparison with Dutch reference population

	SF-36			EQ-5D	
Domain	Reference	UMCU		Reference	UMCU
Physical function	83.0 (22.8)	71.5 (30.3)^a^	Dutch tariff	-	0.68 (0.26)
Social function	84.0 (22.4)	74.5 (27.6)^a^	UK tariff	0.88 (0.19)^c^	0.64 (0.30)^a^
Role physical	76.4 (36.3)	60.9 (32.9)^a^			
Role emotional	82.3 (32.9)	72.5 (30.5)^a^			
Mental health	76.8 (17.4)	72.3 (19.9)^a^			
Vitality	68.6 (19.3)	60.8 (21.3)^a^			
Bodily pain	74.9 (23.4)	72.8 (27.1)^a^			
General health	70.7 (20.7)	61.0 (21.4)^a^			
PCS^b^	50.0 (10.0)	45.6 (10.1)^a^			
MCS^b^	50.0 (10.0)	47.2 (11.1)^a^			

^a^significantly different from reference population

^b^PCS physical component summary, MCS mental component summary

^c^Weighted Dutch reference population, based on the United Kingdom tariff

A total of 1647 patients (88%) reported a problem in one or more domains of the EQ-5D. The index value of our trauma population, calculated using the UK tariff, was significantly lower compared to the Dutch reference population (Table 2).

### Association between trauma and patient characteristics and HRQoL

An overview of the association between each parameter and the subdomains of the SF-36 and the EQ-5Di value is demonstrated in Table 3.

**Table 3:** Univariable linear regression analyses with HRQoL as dependent variable (unstandardized coefficients)

	Physical function	Social function	Role physical	Role emotional	Mental health	Vitality	Bodily Pain	General health	Physical component summary	Mental component summary	EQ-5Di
Age (years)	−0.6 †	−0.2 †	−0.5 †	−0.3 †	−0.04	−0.1 †	−0.2 †	−0.03	−0.2 †	−0.001	−0.003 †
Age categories ‡	−9.7 †	−3.1 †	−7.9 †	−4.3 †	−0.5	−1.6 †	−3.6 †	−0.4	−2.8 †	0.1	−0.04 †
Female gender ‡	−11.9 †	−8.0 †	−10.2 †	−6.2 †	−3.7 †	−7.1 †	−7.7 †	−0.8	−3.6 †	−1.8 †	−0.1 †
Injury type ‡	15.2 †	4.4	10.8 *	4.7	0.4	3.7	4.0	−2.0	3.9 †	−0.3	0.06 *
ISS	−0.3 †	−0.3 †	−0.4 †	−0.3 †	−0.06	−0.1 †	−0.1	0.06	−0.08 *	−0.05 *	−0.002 †
RTS	1.6 *	1.9 *	3.0 †	1.8 *	0.8	1.3 *	−0.6	−0.05	0.3	0.6 *	0.01
Multitrauma	−3.5 †	−3.8 †	−6.2 †	−5.0 †	−0.9	−2.4 *	−0.3	0.06	−0.8	−1.1 *	−0.02
Head AIS ≥ 3	0.05	−2.5	−3.7 *	−4.7 *	−0.4	−1.1	3.6 *	1.5	0.6	−1.2 *	0.003
Face AIS ≥ 3	5.8	2.4	1.0	−4.4	−0.6	0.2	0.6	6.4	2.4	−1.1	0.03
Thorax AIS ≥ 3	−2.7	−3.1	−5.0 *	−1.0	−0.4	−2.0	−2.3	−0.2	−1.2 *	−0.3	−0.03 *
Abdomen AIS ≥ 3	−1.0	−2.0	−2.7	−0.6	−1.1	−1.1	−4.9	1	−0.8	−0.4	−0.03
Extremities AIS ≥ 3	−12.7 †	−3.9 *	−9.4 †	−1.4	−0.2	−2.3 *	−6.5 †	−0.04	−4.1 †	0.9	−0.1 †
Probability of survival ‡	−9.8 *	−12.0 *	−18.9 †	−10.0 *	−2.0	−5.1	−2.9	4.7	7.7 †	3.6	−0.1 *
ICU admission	−6.2 †	−5.0 *	−9.5 †	−5.7 *	−1.9	−3.5 *	−0.3	0.9	−1.5 *	−1.4 *	−0.1 †
H-LOS	−0.4 †	−0.2 †	−0.4 †	−0.3 †	−0.1 †	−0.1 †	−0.2 †	0.02	−0.1 †	−0.04 *	−0.003 †
ICU-LOS	−0.7 †	−0.2 *	−0.7 †	−0.3 *	−0.1	−0.1	−0.1	0.1	−0.2 †	−0.02	−0.005 †

‡ Age categories (years): 1 = 18–40, 2 = 41–60, 3 = 61–70, 4= > 70; Injury type, 1 = blunt 2 = penetrating; ‡ Dichotomous variable: 0 = ps ≥ 0.5, 1 = ps < 0.5

* *p* < 0.05; † *p* ≤ 0.001

In almost all subdomains the female patient had a significantly lower score compared to the male patient. Patients with blunt trauma had a substantially lower physical function.

An increase with one unit of the variables age, ISS, H-LOS, ICU-LOS, RTS and probability of survival showed a significant decrease in the HRQoL in the majority of the subdomains. Age, categorised in four different groups had a large significant influence on the HRQoL. In comparison with the other three age groups the eldest patient group (>70 years) had a lower HRQoL up to 29.1 points in the physical function.

Being severely injured was negatively associated with the HRQoL. A severe injury of the extremities influenced the HRQoL substantially, in particular in the ‘physical function’ domain (12.7 points). The head injury severity had a negative relation with the subdomains ‘social function’, the ‘role physical’, and ‘role emotional’. A notable observation is that the severity of head injury establishes an increase of the subdomain ‘bodily pain’. The other severity of the AIS regions did not have a relationship with the majority of the subdomains of the SF-36 and the EQ-5Di value. An admission to ICU showed to have a predominantly negative relation with the HRQoL. Patients with a predicted death had a significant worse HRQoL outcome in the majority of the subdomains of the SF-36 and the EQ-5Di.

The majority of the significant variables in the subdomains also had an association with the summary components, PCS and MCS. Though the effect on the HRQoL was less expressive.

## Discussion

This study provides an overview of parameters significantly associated with the HRQoL in a general trauma population. Factors associated with HRQoL were age, gender, ICU admission, probability of survival, injury type and severe injury to the head or extremities. These parameters can be used in a model for prediction purposes. Observed and expected HRQoL can be compared and used to evaluate the performance of a trauma centre.

This study provides detailed information which specific subdomain is responsible for the change in the overall HRQoL and to what extent. In literature, other studies only used the summary component of the physical and mental health [23, 24].

A remarkable observation in the present study was that the severity of an injury measured with AIS score did not show a high association with the majority of the subdomains and the PCS and MCS score. Only a severe injury of the extremities and head (AIS ≥ 3) had a highly significant and clinically relevant influence on the HRQoL subdomains. An explanation for this could be the disability in patients with a severe injury of the extremities. For example, pain symptoms after surgery or malunion fractures or disabilities after amputations. Also severe head injuries could cause a lot concentration and mental problems which affect a life substantially. These results correspond with two previous studies which investigated the association of patient characteristics and HRQoL [23, 24]. The authors also concluded that the severity of injury was not associated with the HRQoL outcome.

Gender and age showed to have a substantial influence on the HRQoL in this study. Previous studies demonstrated a difference between gender and age in different reference populations [22, 25]. Therefore, the relation between age and gender with the HRQoL could be overestimated in this study and not only trauma-related.

The ‘age category’ variable in this study shows that a category variant is significantly more associated with the outcome than the continuous variant of the variable. This also accounts for the continuous variable of the admission days and ISS. The increase or decrease of one unit is only a very small change on the large continuous scale. For example, a patient has to be admitted in the hospital for 10 days in order to lower the HRQoL with 4 points. A category variant of these variables might therefore be of more value in a prediction model.

The results of this study could also be used for early intervention programs in patients at risk for a significant decrease of HRQoL in a certain subdomain. For example, physiotherapy or occupational therapy in patients with impairments in the physical domains, and early start of psychotherapy or cognitive therapy in patients with affected mental health.

In current trauma care performance studies survival probabilities are calculated, observed and expected survival evaluated and compared to an international standard [26]. There is a general consensus that nonfatal outcomes should actually also be used for these comparisons, for example quality of life [5, 27–30]. This study identified several factors associated with HRQoL. The next step is to standardise HRQoL outcome for each type of injury and patient, which however is quite challenging due to lack of a gold standard. Another huge challenge is dealing with the fact that HRQoL is influenced by numerous cultural-specific factors, e.g., country specific (luxury) standards. A solution could be the performance of an international consensus-based study. Experts should then agree on the expected HRQoL outcome after a certain injury. This should be specified for a specific type of patient and specific geographical area. There should also be agreement on the acceptable deviation from this norm and on the timing for HRQoL measurements after a trauma.

If HRQoL is to be used as benchmark parameter one of the most important issues to address is which differences in HRQoL are clinically relevant. In the original Medical Outcomes Study published in 1989 by Stewart et al. a 3 to 5 point shift was considered to be an important difference on the SF-36 scale of 1–100 [31]. More recent studies showed that a difference of 6–8.5 points [32] and 12–17 points [16] was required for a clinical relevant difference. These findings show at least that there is no general consensus in the literature regarding this subject. For the EQ-5Di a difference of more than 0.032 points is considered clinically relevant [12]. This seems very little, but the range of the EQ-5Di is very small (−0.33–1.0) and the changes of the index value very minimal (≥0.01).

The SF-36 and EQ-5D were used in this study because these generic instruments are widely used in trauma patients. Still, we have to consider that other trauma specific questionnaires might be necessary for prediction purposes, such as the Trauma Outcome Profile [33] and the recently developed Trauma-Specific Quality of Life questionnaire [34]. Although these trauma-specific instruments should first be evaluated and validated in several different countries in order to use the surveys for prediction purposes.

Some limitations regarding this study need to be addressed. The relatively long follow up time in this study (1–7 years) may have caused other factors apart from the initial trauma or newly developed diseases to have influenced the HRQoL outcome. It is known that HRQoL is also associated with other factors, such as compensation and coping mechanisms [23, 35]. Furthermore, there were some differences between the eligible and participating population which might have influenced the results.

## Conclusion

This study reveals several factors associated with the HRQoL after trauma. These factors were age, gender, ICU admission, injury type, probability of survival and being severely injured, in particular to the head and extremities. The parameters can be used to predict the HRQoL of patients and compare the difference with the observed HRQoL outcome. Future studies must determine the appropriate HRQoL instrument for this purpose and the ideal timing for the measurement. Furthermore, country-specific standardised HRQoL outcomes should be developed in an international consensus-based study. These steps must be taken, in order to further explore the feasibility to use HRQoL as an indicator for trauma centre performances.

## Abbreviations

AIS: Abbreviated injury scale
EQ-5D(i): Euroqol-5 Dimension (index)
GCS: Glasgow coma score
H-LOS: Hospital length of stay
HRQoL: Health Related Quality of Life
ICU-LOS: Intensive Care Unite length of stay
ISS: Injury severity score
MCS: Mental component summary
PCS: Physical component summary
RR: Respiratory rate
RTS: Revised Trauma Score
SBP: Systolic blood pressure
SF-36: The Short Form (36) Health Survey
UMCU: University Medical Center Utrecht
